# Comparing Radiation Dose from Conventional Fluoroscopy to Intraoperative Cone Beam CT (O-arm) during Percutaneous Lesioning Procedures of the Gasserian Ganglion

**DOI:** 10.7759/cureus.345

**Published:** 2015-10-07

**Authors:** Sohum Desai, Vishal J Patel, Rishi R Lall, Daniel Branch, Achal P Patel, Randall Z Allison, David Paulson, Juan R Ortega-Barnett

**Affiliations:** 1 Division of Neurosurgery, University of Texas Medical Branch at Galveston; 2 Neurosurgey, Swedish Neuroscience Institute

**Keywords:** trigeminal neuralgia, cone beam ct, radiation dose, fluoroscopy, percutaneous trigeminal rhizotomy

## Abstract

*Introduction: *The use of intraoperative CT-guidance during the percutaneous treatment of trigeminal neuralgia has become increasingly popular due to the greater ease of foramen ovale cannulation and decreased procedure times. Concerns regarding radiation dose to the patient, however, remain unaddressed. We sought to compare the emitted radiation dose from fluoroscopy with intraoperative CT for these procedures.

*Methods:* A retrospective review of percutaneous lesioning procedures for trigeminal neuralgia performed between 2010 until 2012 at our institution was conducted and radiation doses to the patient were recorded. We subsequently simulated four separate percutaneous trigeminal rhizotomies using the O-arm intraoperative CT (Medtronics, Minneapolis, MN, USA) to cannulate the foramen ovale bilaterally in two formalin-fixed cadaver heads.

*Results:* Seventeen successful percutaneous treatments for trigeminal neuralgia were performed during the study period. Eleven procedures containing complete records were included in the final analysis. For procedures using fluoroscopy, the mean dosage was 15.2 mGys (range: 1.15 - 47.95, 95% CI 7.34 – 22.99). Radiation dosage from the O-arm imaging system was 16.55 mGy for all four cases. An unequal variance t-test did not reach statistical significance (p=0.42).

*Conclusions:* We did not observe a significant difference in radiation dose delivered to subjects when comparing CT-guided foramen ovale cannulation relative to fluoroscopy for percutaneous lesioning of the Gasserian ganglion. Additional study is required under operational settings.

## Introduction

Trigeminal neuralgia (TN) classically presents with unilateral paroxysmal electric, shock-like facial pain in the distribution of the trigeminal nerve [1]. The annual incidence of TN is reportedly 4-13 per 100,000 individuals and is one of the most frequent neuralgias of the elderly [2-3]. Patients are typically over 50 years old with a slight female predilection. Pain originates in the right trigeminal distribution more often than left (60% vs. 40%) and typically involves both the maxillary and mandibular nerves followed by only the maxillary nerve (42% and 20% of cases, respectively) [4-5].

For patients who fail medical therapy, potential treatments for TN include ablative and non-ablative therapies of the trigeminal nerve. Microvascular decompression is the only non-ablative physiologic treatment of trigeminal neuralgia in medically suitable candidates without radiographic or clinical evidence of neoplastic or demyelinating conditions. Often, these patients are found to have vascular compression of the root entry zone of the trigeminal nerve secondary to aberrant arterial loops or veins. Ablative procedures are those involving lesioning or destruction of the trigeminal nerve or the Gasserian ganglion. This category of procedures includes stereotactic radiosurgery, balloon rhizotomy, glycerol rhizotomy, and radiofrequency thermocoagulation. The last three are performed percutaneously through the foramen ovale. These procedures are preferred in patients who have trigeminal neuralgia secondary to demyelinating conditions, who are medically unsuitable for surgery, or in whom prior microvascular decompression has failed.

A percutaneous trigeminal rhizotomy (PTR) through the cannulation of the foramen ovale was originally described by Härtel in 1911 [6]. Traditionally, this is accomplished with the assistance of intraoperative C-arm fluoroscopy. Recently, however, several studies have reported successful foramen ovale cannulation using intraoperative CT navigation as well [7-8]. Reported benefits of CT-guidance include better spatial orientation, fewer unsuccessful attempts at cannulation, and shorter procedure length [8]. These advantages, however, have been tempered by concerns regarding increased radiation dose to the patient relative to traditional fluoroscopy [9]. The induction of future cancers, such as myeloma, leukemia, lung cancer, thyroid cancer, breast cancer, bone cancer, and skin cancer, as well as hereditary effects, shortening lifespan, and cataract formation in the eyes are associated with ionizing radiation as a function of the total radiation dose [10-11].

No previous studies have compared radiation doses using intraoperative CT versus fluoroscopy for percutaneous trigeminal rhizotomy. The goal of this study was to assess whether intraoperative cone-beam CT is associated with increased patient radiation dose relative to traditional fluoroscopy for patients undergoing PTR.

## Materials and methods

The University of Texas Medical Branch Institutional Review Board approved this study (approval #11-003). Informed consent was obtained from all patients in accordance with the guidelines of the institutional review board. A retrospective review of all percutaneous lesioning procedures for trigeminal neuralgia performed between 2010 till 2012 at UTMB was conducted. The radiation dosing records from these cases were obtained and analyzed.

This procedure was typically performed by either a trainee (PGY-5) or an attending.

The dose represents an estimate of radiation exposure to the patient’s skin by calculating the dose 10 cm proximal to the isocenter of the beam and fluoroscopy time. At our institution, this is periodically verified for accuracy with a phantom device.

In the second part of the study, we simulated four cannulations of the foramen ovale using two formalin-fixed cadaver heads, once on each side. A noninvasive landmark FESS strap with an attached reference frame was first secured. Next, a high-definition 3D scan of the skull base was obtained using the Medtronic O-arm (Minneapolis, MN, USA). These images were uploaded to the Medtronic Stealth S4 Station (Minneapolis, MN, USA) and used to cannulate the foramen ovale. Dosing information was then recorded.

Statistical analysis was performed using Microsoft Excel (2010). The differences were considered to be significant if the statistical P-value was < 0.05.

## Results

A total of seventeen treatments occurred in thirteen patients during the study period. Radiation dosing and demographic information was not available in six procedures. The final analysis included eleven treatments performed on five females and three males (Table 1). These patients ranged in age from 44 to 92 years (average age of 64 years).

**Table 1:** Demographic, Clinical Presentation, and Radiation Dosage of Patients Undergoing Percutaneous Procedures for Trigeminal Neuralgia

Patient	Age (Years)	Gender	Procedure	Radiation Dose (mGy)	Attempts	Surgeon
Patient #1	66	Female	Blockade	9.92	1	Attending
	67	Female	Blockade	28.26	1	Attending
Patient #2	62	Female	Blockade	7.84	1	Trainee
	62	Female	Radiofrequency ablation	10.37	1	Trainee
	62	Female	Radiofrequency ablation	19.39	1	Trainee
Patient #3	92	Male	Radiofrequency ablation	13.48	1	Trainee
Patient #4	62	Female	Blockade	10.54	1	Trainee
Patient #5	54	Male	Radiofrequency ablation	15.52	2	Trainee
Patient #6	44	Female	Radiofrequency ablation	1.15		
Patient #7	60	Female	Radiofrequency ablation	47.95		
Patient #8	70	Male	Radiofrequency ablation	2.4		

Four procedures were PTR blockade via ethanol injection and seven were PTR radiofrequency ablation. There were no procedural complications in this series.

In a majority of procedures (6 of 11 cases), cannulation was performed solely by trainees. A single attempt resulted in successful cannulation in 7 of 11 cases. One case required two attempts. Data regarding the number of attempts and identity of the surgeon was unavailable in the remaining three cases.

For procedures using fluoroscopy, the average dosage was 15.2 mGys (range: 1.15-47.95, 95% CI 7.34 – 22.99). Radiation dosage from O-arm imaging system was 16.55 mGy for all four cases. An unequal variance t-test did not reach statistical significance (p=0.42).

## Discussion

Neuronavigation is frequently used during cannulation of the foramen ovale due to morbidity and mortality associated with the transovale approach of the Gasserian ganglion (Table 2).

**Table 2:** Literature Regarding Use of Navigation to Cannulate Foramen Ovale

Author	Study Type	Patients	Guidance	Conclusions
Bohnstedt, 2012	Case series	4	O-arm, spine reference frame attached with a FESS strap, Synergy software	Useful for difficult-to-access formen ovale
Lin, 2011	Case-controlled series	42	BrainSUITE iCT	Shorter length of time (p=0.011), fewer cannulation attempts
Yang, 2010	Case-controlled series	79	CT fluoroscopy	Shorter length of time compared to conventional fluoroscopy (p=0.001)
Koizuka, 2010	Case series	13	CT fluoroscopy	Procedure length 10- 31 secs. Improved pain scores in 11 patients
Mandat, 2009	Case series	3		Pain relief at 1 year in all 3 patients
Bale, 2006	Case series with cadaveric component	15	VBH mouthpiece, NECT, EasyGuide, Stealth Station Treon	
Liu, 2005	Case series	18	3D CT	No complications. Pain relief in 13 patients.
Gusmão, 2003	Case series	10	CT fluoroscopy	Cannulation time in all patients was less than 40 seconds. Radiation exposure is less than fluoroscopy but greater than sequential CT.

The C-arm has traditionally been used to navigate the needle tip towards the intersection of the top of the petrous bone with the clivus using Härtel's technique of utilizing the cheek point, zygomatic point, and mid-pupillary point as anatomical guides [6]. Utilizing lateral fluoroscopy for navigation to the foramen ovale allows for only two-dimensional images and requires the surgeon to utilize the aforementioned landmarks during a percutaneous transovale approach. Cone beam CT provides three-dimensional image guidance in which the patient's head can be adjusted perioperatively due to the lack of pin fixation [7, 12].

Three-dimensional mapping with guided instrumentation was developed and popularized in spine surgery. These features of O-arm use also offer many benefits in the cannulation of the foramen ovale. Three-dimensional imaging affords the surgeon the capability of a single path cannulation, thereby avoiding the buccal vessels, intracranial vessels, and limiting infectious risks [7]. O-arm guidance has the capability of lowering the learning curve in novice surgeons attempting the cannulation of the 8 x 4 mm foramen ovale [13-14]. Cannulation of the carotid artery canal, jugular foramen, inferior orbital fissure, and Vesalius foramen have been described during attempted foramen ovale cannulation, resulting in adverse events, including cavernous sinus puncture, optic nerve injury, and temporal lobe hematoma [15-16]. Single path cannulation also decreases the operative time and anesthesia exposure, as does the lack of a necessary X-ray technician to utilize the O-arm (required for C-arm) and a decreased likelihood of field contamination by rotation of the undraped portions of the C-arm [7, 17].

Bohnstedt, et al. has described the efficacy of the O-arm use in PTR for the treatment of TN originally developed for perioperative imaging during spine surgery. Multiple case series have shown the transovale approach utilizing O-arm to be well tolerated with an efficacy equal to or better than that of C-arm guided approaches [1, 7-8, 12-13, 18]. This approach may be specifically useful in difficult to treat cases due to limited access of the foramen ovale [7]. By decreasing the operative time and retaining intraoperative accuracy, the O-arm has been shown to provide superior perioperative imaging during the percutaneous approach of the foramen ovale while limiting radiation exposure to the surgeon [7-8, 13].

One concern in utilizing O-arm imaging is the increased radiation exposure to the patient during PTR. In our study, we found radiation exposure to the patient in fluoroscopic (15.2 mGy) and O-arm-guided (16.55 mGy) cannulation of the foramen ovale to be equivalent (p=0.4) and, therefore, not exposing the patient to any increased radiation dose compared to that routinely given by C-arm imaging.

## Conclusions

Radiation dosing using fluoroscopy and O-arm image guidance were not significantly different in our study. Therefore, the percutaneous approach to the foramen ovale using the O-arm seems to be a safer and faster procedure than a fluoroscopy-guided approach since it limits radiation exposure to the surgeon and does not significantly increase radiation exposure to the patient.

Limitations of this study include both the sample size and the retrospective study design, as well as the use of cadaver rather than live subjects to measure radiation exposure from O-arm imaging. Future investigations should include large, prospective studies utilizing live patients. Measurement of radiation dosing given to different structures, including the cornea and thyroid gland, would be beneficial to investigate radiation exposure to different areas between C-arm fluoroscopy and O-arm use.
